# A129 CLINICAL RELIABILITY OF LOWERED ANTI-TISSUE TRANSGLUTAMINASE LEVEL FOR SEROLOGIC DIAGNOSIS OF PEDIATRIC CELIAC DISEASE

**DOI:** 10.1093/jcag/gwae059.129

**Published:** 2025-02-10

**Authors:** A Lang, M Wiepjes, M Zarrabi, D M Isaac, J Turner

**Affiliations:** University of Alberta, Edmonton, AB, Canada; Dalhousie University, Halifax, NS, Canada; Alberta Health Services, Edmonton, AB, Canada; University of Alberta, Edmonton, AB, Canada; University of Alberta, Edmonton, AB, Canada

## Abstract

**Background:**

The most recent North American pediatric gastroenterology guidelines on diagnosing celiac disease (CD) published in 2016 recommend endoscopic diagnosis (ED) with histologic evaluation as the gold standard in all patients. This contrasts with European guidelines, which have advocated for a serologic diagnosis (SD) in eligible patients, since 2012. The Stollery Children’s Hospital (Edmonton, Alberta), has incorporated SD following internal validation since 2016. Until 2020, SD used an anti-tissue transglutaminase (aTTG) >200 IU/mL with confirmatory HLA typing. After 2020, SD used aTTG >10x the upper limit of normal (ULN) on 2 separate blood-draws.

**Aims:**

The aim of this study was to determine the clinical reliability of a lowered threshold of aTTg >5x ULN for SD in our centre. A secondary aim was to determine estimated healthcare cost savings if this change were implemented.

**Methods:**

We completed a retrospective chart review on pediatric patients (<18 years) diagnosed with CD at our centre from 2018-2021. Patients who underwent endoscopy for suspected CD were included. The costing method was a mix of aggregate costing for endoscopy performed in ambulatory care of the hospital plus micro-costing of other service utilizations such as physician fee for endoscopy, anesthesiologist fee, lab test and processing fees. To calculate the estimated cost savings, we subtracted the estimated costs of diagnosis in our population when all those meeting SD avoided endoscopy from the estimated total cost of diagnosis if these patients had all undergone ED.

**Results:**

In total, 507 patients underwent endoscopy (Figure 1). In patients with aTTG >10x ULN, specificity for CD diagnosis was 99.2% with positive predictive value (PPV) 98.5%. In patients with aTTG >5x ULN, specificity remained 99.2% with PPV 99.5%. There was one false positive case (aTTG 104 IU/L, negative biopsies).

In our study population, use of lowered SD threshold from 2020-2021 would lead to 34 (10.8%) fewer endoscopies, with associated healthcare cost savings of an estimated $88,919 (Table 1).

**Conclusions:**

In our centre, a lower cut-off value for SD may improve time to diagnosis and initiation of a gluten-free diet, and is cost saving. Preliminary results show comparable performance to the currently accepted diagnostic approach used at our centre. Future directions include validating these findings with a larger sample size, as well as determining acceptance of this diagnostic method by patients and families, given the rare but real potential for false positive diagnoses.

Estimated Cost Savings of ED vs SD

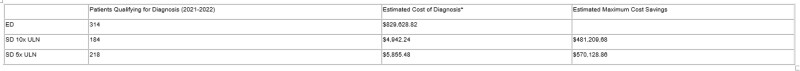

*Average cost of ED= $2642.13, Estimated cost of SD = $26.86

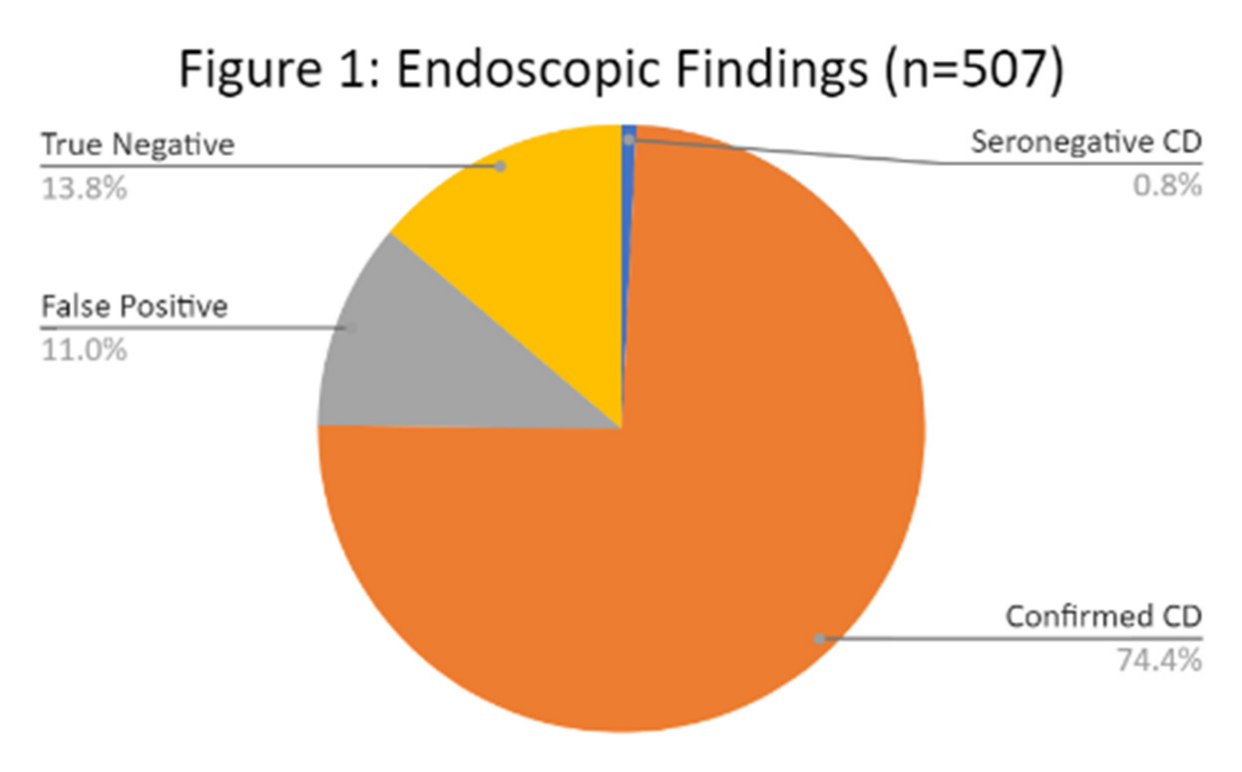

**Funding Agencies:**

None

